# Impact of progesterone-free luteal phase support following natural cycle frozen embryo transfer: Study protocol for a multicenter, non-inferiority, randomized controlled trial

**DOI:** 10.3389/fmed.2022.1014946

**Published:** 2022-11-15

**Authors:** Wen-Jing Jiang, Zhen-Gao Sun, Jing-Yan Song

**Affiliations:** ^1^The First Clinical College, Shandong University of Traditional Chinese Medicine, Jinan, China; ^2^Reproductive Center of Integrated Medicine, The Affiliated Hospital of Shandong University of Traditional Chinese Medicine, Jinan, China

**Keywords:** luteal phase support (LPS), natural cycle, frozen embryo transfer (FET), spontaneous ovulation, assisted reproductive technology (ART)

## Abstract

**Introduction:**

Nowadays, frozen-thawed embryo transfer (FET) has become one of the standard treatments for infertility in the field of assisted reproductive technology (ART). Natural cycle FET (NC-FET) has many advantages, such as simplicity and economics, no effect on patients’ menstrual cycles, estrogen and progesterone levels, as well as no interference in endometrial growth and transformation, which is aligned with the natural physiological state of embryo implantation. Nonetheless, there is a controversy regarding the need for luteal phase support (LPS) during NC-FET cycles. The purpose of this study is to assess whether LPS was not inferior to non-LPS in terms of OPR in NC-FET cycles.

**Methods and analysis:**

This study including 1,010 ovulatory women undergoing *in vitro* fertilization (IVF)/intracytoplasmic sperm injection (ICSI) cycles with an elective freeze-all strategy followed by NC-FET will be performed at four university-affiliated reproductive centers. Participants will be randomly assigned in a 1:1 ratio to receive LPS treatment or not. This study is designed as an open-label, non-inferiority, randomized controlled trial (RCT), and the primary statistical strategies were intention-to-treat (ITT) and per-protocol (PP) analysis.

**Discussion:**

There may not have been any significant difference in the chance of a live birth after FET if no progesterone was supplemental during the luteal phase. However, due to the limited number of previous studies, which are mainly retrospective, evidence is still limited. Thus, by conducting this multicenter RCT, we intend to evaluate whether LPS is necessary in NC-FET.

**Ethics and dissemination:**

A Reproductive Ethics Committee of the Affiliated Hospital of Shandong University of Traditional Chinese Medicine (SDUTCM) has approved this study. This study will handle the data as required by general data protection regulations. Participants will sign a written informed consent regarding participation in the study and storage of blood samples in a biobank for future research. This study will be monitored by study personnel trained in Good Clinical Practice who are not involved in the study. The results of this study will be disseminated through publication in international peer-reviewed scientific journals.

**Clinical trial registration:**

[https://www.chictr.org.cn/], identifier [ChiCTR2200057498].

## Highlights

- The first clinical trial to demonstrate that NC-FET without LPS can be non-inferior to NC-FET with LPS, minimizing the pain and need for medication.
- The purpose of this study is to provide more evidence-based support through a multicenter, non-inferiority, randomized controlled trial.
- Physicians and participants could not be blinded to treatment allocation.

## Introduction

In recent years, with the significant improvement of ovarian stimulation regimens and embryo cryopreservation and thawing techniques, the frozen-thawed embryo transfer (FET) procedure has gained more popularity in assisted reproductive technology (ART) than fresh embryo transfer (1). Additionally, it has been shown that FET is effective in reducing the incidence of ovarian hyperstimulation syndrome (OHSS), preserving remaining embryos, and increasing the cumulative live birth rate (LBR) (2, 3). In general, NC-FET is indicated for patients who experience regular ovulation (4, 5). The NC-FET is cost-effective, does not affect the menstrual cycle, does not interfere with estrogen and progesterone (P_4_) levels, or endometrial growth and transformation, and conforms to the natural physiological state of embryo implantation, despite the disadvantages of repeated monitoring and high cancelation rates (6). Furthermore, a recent large retrospective study involving 14,421 cycles demonstrated a lower early pregnancy loss rate with NC-FET, while the LBR was higher.

In NC-FET cycles, the necessity of luteal phase support (LPS) is still controversial. An investigation of 84 UK IVF clinics revealed that, in natural FET cycles, 31% administer LPS always, while 44% administer it sometimes (7). In another online survey of 179 IVF clinics around the world (representing an estimated 39 thousand FET cycles annually), it was found that more than half (57%) of clinics are using LPS in natural FET cycles, and 49% of clinics are using P_4_ exclusively (8). Despite common use, LPS is not universally used in natural FET cycles, as both surveys underscore. In 2013, a RCT with small sample size (*n* = 102) conducted by Eftekhar et al. comparing NC-FET with no LPS, LPS did not result in higher clinical pregnancy and implantation rates or lower miscarriage rates (9). Coincidentally, Waldman et al. reported similar results in their retrospective study (10). Nevertheless, several recent studies showed that patients undergoing LPS with vaginal P_4_ in NC-FET had higher LBR and lower miscarriage rates (11–13). In addition to LPS with P_4_, patients who received LPS by intramuscular human chorionic gonadotropin (hCG) had a higher ongoing pregnancy rate (OPR) in NC-FET, whereas the RCT results of Lee et al. indicated that this LPS protocol did not exert a similar effect on the OPR (14, 15). Recent meta-analyses indicated a positive impact of P_4_ supplementation after FET in natural cycles (16–18), but further large RCTs are required to confirm these findings.

Therefore, it is still unclear whether or not we should perform LPS in NC-FET. Additionally, previous studies still have limitations such as single center, small sample size, large population heterogeneity, different types, routes and times of P_4_ administration, and inconsistent study endpoint settings. Thus, to determine whether LPS affects reproductive outcomes in NC-FETs, we conducted this well-designed RCT.

## Methods and analysis

### Study design

**FIGURE 1:**
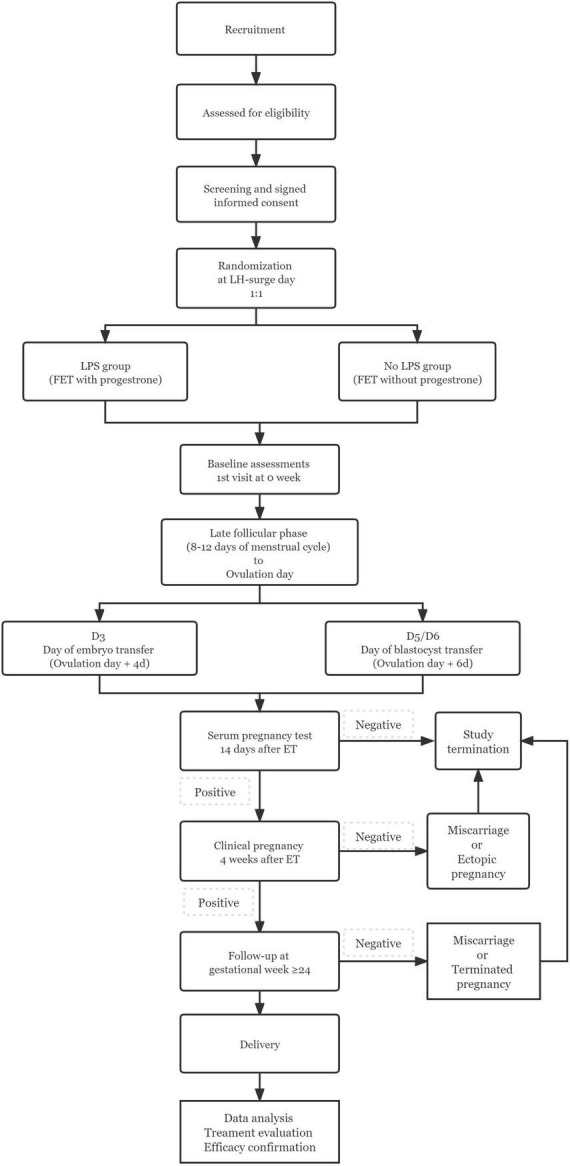
Study flow chart.

The study is designed as an open-label, non-inferiority RCT, including reproductive centers of four tertiary care hospitals in mainland China. The flow chart and study process schedule are shown in Figure 1 and Table 1.

**TABLE 1:** Overview of study visits.

	Baseline * (2–4 days at menstruation)	Late follicular phase (8–12 days of menses) –ovulation day	D3 of embryo transfer (ovulation day + 4 days)	Blastocyst transfer (ovulation day + 6 days)	Day of pregnancy test (ET day + 14 days)	Pregnancy follow-up	Follow-up 1 year
Information and counseling	*						
Signing of informed consent	*						
Treatment-related data collection	*				*	*	*
Randomisation	*						
Transvaginal ultrasound scan	*	*				*	
Blood sample	*	*	*	*	*		
Quality of life questionnaire						*	

*All participants.

### Objectives

At present, there is no clear evidence to suggest that LPS is required for NC-FET cycles following spontaneous ovulation. Therefore, there is still a fierce debate in clinical practice about whether patients treated with NC-FET should receive LPS. Our hypothesis is that no LPS is not inferior to OPRs in patients treated with LPS.

### Eligibility criteria

#### Inclusion criteria

Having regular ovulatory cycle; at least one embryo or blastocyst available for transfer.

#### Exclusion criteria

Age ≥ 45 years old, body mass index (BMI) ≥ 30 kg/m^2^; oocyte donation cycle; chromosomal abnormalities in both or one of the couples.; egg donation cycle; history of pelvic chemoradiotherapy; polycystic ovarian syndrome (PCOS); ovarian endometrioma, uterine fibroids, endometrial polyps or intrauterine adhesions requiring surgical treatment; history of repeated implantation failure (Infertile patients < 40 years old experience ≥ 3 oocyte retrieval cycles, and fresh or FETs cumulatively transfer at least four high-quality embryos without clinical pregnancy) and recurrent pregnancy loss (two or more pregnancy losses). Patients may withdraw from the study at any time without reason.

### Study population and recruitment

The study population include patients undergoing elective freeze-all strategy following IVF/ICSI cycles. Morphological evaluation of cleavage-stage embryos and blastocysts will be performed according to the Racowsky et al. **(19)** and the Gardner Scoring System **(20),** respectively. At least one grade I or II embryo with D3 blastomeres > 6 or high-quality blastocysts with a score ≥ 3BB will be cryopreserved. The investigators will contact all patients interested and eligible for the study by telephone. Patients interested in participating in the study will visit the corresponding reproductive center on days 2–4 of the menstrual cycle, after having fully understood the content of the study. Here, they further understand the details of the study and sign an informed consent. Each individual may be included only once and only in the first FET cycle after the oocyte retrieval. All medical personnel in the study will receive all necessary information and training to uniformly handle patients at each reproductive center. All sites staff have sufficient experience in conducting clinical trials.

### Randomization

Randomization will be performed by an independent statistician using a computer-generated randomization schedule with block randomization (block size of four) stratified by female age (< or ≥ 35 years) at LH-surge day in NC-FET cycle. Patients will be randomized in a 1:1 ratio to one of the following groups: (i) LPS group: Patients will receive vaginal combined intramuscular P_4_ treatment starting on the day of ovulation. (ii) Non-LPS group: Neither LPS nor any other therapies are administered to patients.

### Interventions

Frozen-thawed embryo transfer will be performed in NC with spontaneous ovulation. Transvaginal ultrasonography (TVUS) is used to monitor follicular development and endometrial growth. In the late follicular phase, that is, on days 8–12 of the cycle (depending on the length of the patient’s menstrual cycle), when the dominant follicle develops to a mean diameter of more than 14 mm, serum LH level is monitored every other day until the LH surge appeared (21). When the endometrial thickness reaches more than 8 mm and the endometrium is classified as type A or B, embryo transfer is performed 4 days (D3 embryos) or 6 days (D5/D6 blastocysts) after the appearance of the LH surge. If luteinization of unruptured follicles occurs, embryo transfer is canceled. Plasma hCG levels were measured 14 days after embryo transfer.

Patients in the LPS group are given P_4_ sustained-release gel Crinone ^®^ 8% (Crinone, Merck Serono, Switzerland) vaginally, 90 mg/time, once a day and P_4_ injection (Zhejiang Xianju Pharmaceutical Co., Ltd., Zhejiang, China) intramuscularly, 20 mg/vial, twice a day until 14 days after FET (21). For pregnant women, LPS treatment should be continued until 10 weeks of gestation.

### Data collection

Relevant treatment data of patients are collected at the corresponding time points: (1) basic data (2–4 days of menstruation); (2) dominant follicle development to an average diameter of more than 14 mm to the day of ovulation under TVUS monitoring; (3) D3 embryo transfer day (ovulation day + 4 days); (4) blastocyst transfer day (ovulation day + 6 days); (5) pregnancy test day (embryo transfer day + 14 days). In the case of pregnancy and delivery, data will be collected from the patient’s medical records as well as the birth records of the newborn for registration of obstetric and neonatal outcomes up to 1 year after delivery. Any protocol deviations or unanticipated effects on the conduct of the trial will be registered. All study personnel will be trained in data collection and entry, handling of data discrepancies, and procedures performed during study visits. Data collection forms are available by contacting the study steering committee.

### Blood sample collection

As part of the basic data stage, follicle-stimulating hormone (FSH), LH, estradiol (E_2_), and P_4_ should be measured, followed by LH, E_2_, and P_4_ until an LH surge occurs. E_2_ and P_4_ should be measured on the day of embryo transfer, and E_2_, P_4_, and β-hCG should be measured on the day of pregnancy test.

### Research biobank and biobank for future research projects

In addition to samples for serial analysis, a total of 12 ml of blood samples (serum, and plasma) will be drawn at each sampling time and stored in a -20°/-80° freezer at Rigs Hospitalet. Samples will be identified by anonymous subject ID numbers to maintain subject confidentiality. In this study, the sample can be used as a backup of continuous analytical samples or stored in a biological sample bank for possible future research projects. Patients will be asked to sign a separate informed consent form to store the blood samples in a biobank for future research. Additional approval from the Ethics Committee will be required for future projects. If samples are not used, they will be destroyed according to the biomaterial destruction rules after the end of the study or no later than 5 years after the last patient is enrolled.

### Transvaginal ultrasound

During FET cycles, Transvaginal ultrasonography is required as per clinical routine. Transvaginal ultrasonography is used to determine endometrial thickness and antral follicle number on days 2–5 of menstruation in a treatment cycle. Endometrial thickness and the size of the dominant follicle are estimated in the late follicular phase, i.e., on days 8–12 of the cycle depending on the length of the patient’s menstrual cycle. TVUS was repeated until the dominant follicle reached more than 17–18 mm and an LH surge appeared. If conception occurs, an early pregnancy scan will be performed at pregnancy 7–8 to assess fetal viability and crown-rump length.

### Data management

According to the informed consent form signed by all study participants, the study staff and relevant regulatory authorities can directly access the relevant data of patients to study and follow up the relevant conditions of patients and facilitate the quality control. All data for the study will be uploaded into electronic case report forms in the study’s electronic data capture system for ease of data integration and management. The study electronic data capture system had a complete audit trail based on anonymous subject identification numbers used in the study. The system will interval program numeric data to detect possible input errors in the study. This platform is protected by a password-protected access system. The system will automatically backup data daily and store it on the server. Documents containing patient identifying information will be stored separately in a document with limited access. Source documents will be reviewed by Good Clinical Practice-trained study personnel (not participating in the study) to ensure completeness and accuracy of the data. The reviewer will assess the overall quality of the data and confirm that the site meets the protocol requirements. Data will be processed in accordance with the Data Protection Act and approved by the Data Review Center. The principal study site will develop a unified data processing agreement form with other cooperative centers and strictly implement it.

### Data sharing plan

Study data will be shared according to International Committee of Medical Journal Editors guidelines. Data sharing may occur with parties who provide the purpose associated with the detailed use of data. The other party’s study must obtain the corresponding study approval. The study data will not be shared with the same team as the purpose of this study. Data sharing will take place 3 months after the publication of papers involving the study’s primary and secondary outcomes. Any new research project must be conducted under the premise of approval. The party requesting data sharing will be charged accordingly.

### Outcome measures

#### Primary outcome

The primary outcome is OPR. Ongoing pregnancy is defined as intrauterine pregnancy confirmed by TVUS examination at more than 12 weeks of gestation accompanied by normal fetal heart beats.

#### Secondary outcomes

(1) Positive pregnancy: positive pregnancy refers to serum β-hCG ≥ 10 mIU/ml 14 days after embryo transfer; (2) Embryo implantation rate: the number of gestational sacs determined by TVUS examination divided by the total number of embryos transferred; (3) Pregnancy loss: pregnancy loss refers to the loss of an intrauterine pregnancy that is less than 28 weeks gestational age; (4) Ectopic pregnancy: ectopic pregnancy refers to the implantation and development of embryos in sites other than the uterine coelom; (5) Multiple pregnancy: multiple pregnancy refers to the simultaneous presence of two or more fetuses in the uterine cavity; (6) Live birth: live birth refers to newborns with gestational age at delivery ≥ 24 weeks and heartbeat and respiration; (7) Pregnancy-related complications: including preeclampsia, gestational hypertension, cesarean section, and postpartum hemorrhage (>1,000 ml); (8) Obstetric complications: including gestational diabetes (GD), placental pathology (accreta, previa), specify spontaneous preterm birth or induced preterm birth; macrosomia; small for gestational age, large for gestational age; low birth weight: [absolute weight, relative weight compared to mean at specific gestational local reference curve (*p* or Z-value)], and perinatal death.

### Non-inferiority design and power calculation

The present study will adopt the non-inferiority design. Due to previous studies showing that NC-FET treated with LPS does not achieve the same outcome as NC-FET treated without LPS, and that treatment without LPS has the advantages of being less costly and less painful. Specifically, we assumed a 10% margin of non-inferiority and a 50% OPR in two arms based on our center’s experience with FET. The number of cases in each group was calculated at 429, according to the design of 1:1 parallel non-inferiority, the unilateral test with alpha = 0.05, and the power of 90%. Assuming a drop rate of 15%, there are 505 cases per group and 1,010 cases for the two groups.

### Drop-outs and cancel cycles

Dropout refers to the study participants’ voluntary decision to withdraw from the study due to personal subjective factors. The canceled cycles were those who were forced to cancel the cycles due to endometrial lesions found in TVUS, failure of follicular development or failure of embryo thawing and resuscitation up to 21 days in the cycle. The researchers will make a detailed summary of the number and causes of dropout and cancelation in both groups and completion characteristics within and between groups. Based on our experience, the dropout rate will be up to 15%. If the actual dropout rate is higher than expected, we will discuss the potential bias, analyze the differences between the results and draw conclusions accordingly.

### Statistical analysis and interpretation of data

The intention-to-treat (ITT) analysis includes both dropout and cancelation cycles. The per-protocol (PP) analysis includes all patients who strictly followed the study protocol. In the as-treated analysis, patients who subsequently received LPS but not NC-FET are excluded, however, those who subsequently received LPS from NC-FET until 10 weeks of gestation are included. We measured OPRs and identified differences between groups based on relative risk (RR) and 95 confidence interval (CI). PP analyses will be also performed for all reproductive outcomes.

Continuous data were compared using Student’s *t*-test and results are presented as mean (standard deviation, SD) or median (inter-quartile range, IQR). Categorical data with expected frequencies less than five is assessed using χ^2^ analysis and Fisher’s exact test. *P*-values less than 0.05 is considered statistically significant. Data analysis will be performed using SPSS 26.0 and R 4.1.3. Multivariate logistic regression analysis will be performed to identify variables independently associated with OPR.

### Patient and public involvement statement

Patients and the public are not involved in developing research questions or study design. The results of the study will be disseminated to the participants and their families by telephone and the patient’s attending physician.

### Ethics and dissemination

This study has received ethical approval from the Reproductive Ethics Committee of the Affiliated Hospital of SDUTCM (SDUTCM-RM-2022076) and all participating hospitals. The researchers had obtained written informed consent from each patient participating in the study before the start of the study. Any amendments to the protocol that could affect the design, conduct, and safety of the study will be implemented after formal amendment by a committee. Data will be reviewed and approved by an external Data and Safety Monitoring Board. Details of data management will be given elsewhere in this paper.

It is sufficiently assured that the trial personnel’s safety is assured. Whether they are administered LPS after NC-FET make the difference between the two regimens. We don’t expect there to be a difference in the OPR between those who receive LPS and those who do not receive LPS. In most cases, the study will not cause discomfort or harm to the patient. When blood is drawn and P_4_ is injected intramuscularly, the patient may feel pain and discomfort and may experience minor bruising. Neither will participants incur additional financial costs nor will they receive financial compensation for participating in the clinical study.

The study will be presented at national and international scientific meetings by presenting the results in scientific journals and the Chinese Clinical Trial Registry (ChiCTR) and published in high impact peer-reviewed international scientific journals for reproductive medicine. The results of the common interest will be reported in the public media.

## Discussion

At present, there is no clear evidence that LPS is required for NC-FET cycles following spontaneous ovulation. Therefore, Weissman conducted a web survey of FET in 2020 involving 179 IVF centers in 56 countries involving 39152 FET cycles (8). In this survey, it was found that 44% of participants did not administer LPS to patients during NC-FET cycles. At present, relevant studies have the limitations of multi-center, insufficient sample size, different types and routes of P_4_ administration, and different administration time, so larger RCTs are needed for further study. We designed this multi-center large RCT by a group of experienced professionals for this purpose. In our hypothesis, patients without LPS do not experience a lower OPR than those who receive LPS treatment. Provided that the hypothesis is validated, we can minimize the pain and economic burden caused by medication. The results of this study may be implemented clinically immediately after publication. Consequently, we hope that this study will lead to the development of new standards in NC-FETs on both a national and international scale.

## Trial status

The trial was registered on 14 March 2022 (ChiTR). The actual study start date was 1 July 2022 and the expected study end date was 31 December 2023. The enrollment start date was 15 July 2022; the anticipated enrollment end date was 31 May 2023.

## Author contributions

J-YS and Z-GS participated in the conception, design, writing, and editing of the study protocol. W-JJ wrote the first draft. All authors were involved in the critical revision of this manuscript and approved the final version of the manuscript prior to submission.
